# Mechanical wave velocities in acute myocardial infarction: an exploratory study using three-dimensional high frame rate echocardiography

**DOI:** 10.1093/ehjimp/qyaf060

**Published:** 2025-05-15

**Authors:** Marlene Iversen Halvorsrød, Mohammad Mohajery, Torvald Espeland, Sebastien Salles, Asbjørn Støylen, Lasse Løvstakken, Bjørnar Grenne

**Affiliations:** Department of Circulation and Medical Imaging, Faculty of Medicine and Health Sciences, Norwegian University of Science and Technology, PO Box 8905, NO-7491 Trondheim, Norway; Clinic of Cardiology, St. Olav’s Hospital, Trondheim University Hospital, Postbox 3250 Torgarden, NO-7006 Trondheim, Norway; Department of Circulation and Medical Imaging, Faculty of Medicine and Health Sciences, Norwegian University of Science and Technology, PO Box 8905, NO-7491 Trondheim, Norway; Department of Circulation and Medical Imaging, Faculty of Medicine and Health Sciences, Norwegian University of Science and Technology, PO Box 8905, NO-7491 Trondheim, Norway; Clinic of Cardiology, St. Olav’s Hospital, Trondheim University Hospital, Postbox 3250 Torgarden, NO-7006 Trondheim, Norway; Department of Circulation and Medical Imaging, Faculty of Medicine and Health Sciences, Norwegian University of Science and Technology, PO Box 8905, NO-7491 Trondheim, Norway; Department of Circulation and Medical Imaging, Faculty of Medicine and Health Sciences, Norwegian University of Science and Technology, PO Box 8905, NO-7491 Trondheim, Norway; Clinic of Cardiology, St. Olav’s Hospital, Trondheim University Hospital, Postbox 3250 Torgarden, NO-7006 Trondheim, Norway; Department of Circulation and Medical Imaging, Faculty of Medicine and Health Sciences, Norwegian University of Science and Technology, PO Box 8905, NO-7491 Trondheim, Norway; Department of Circulation and Medical Imaging, Faculty of Medicine and Health Sciences, Norwegian University of Science and Technology, PO Box 8905, NO-7491 Trondheim, Norway; Clinic of Cardiology, St. Olav’s Hospital, Trondheim University Hospital, Postbox 3250 Torgarden, NO-7006 Trondheim, Norway

**Keywords:** myocardial infarction, high frame rate echocardiography, 3D echocardiography, mechanical wave, atrial kick wave, myocardial stiffness

## Abstract

**Aims:**

High frame rate (HFR) echocardiography captures myocardial mechanical waves (MWs), reflecting critical tissue properties. The aim was to assess the feasibility of 3D HFR echocardiography for estimating MW velocities in acute myocardial infarction (AMI) patients and to compare MW velocities with those in controls.

**Methods and results:**

Twenty patients with ST-elevation AMI were included within 48 h of reperfusion therapy. 3D high-quality recordings (∼20 volumes/s) were acquired for myocardial segmentation and 3D HFR recordings (750 volumes/s) for measuring the atrial kick wave propagation velocity. MW velocities were compared with 20 controls. MW velocities were successfully measured in 93% of subjects (17 patients and 20 controls). The segmental feasibility was 97%. Global MW velocities were significantly higher in AMI patients than controls (2.1 ± 0.6 m/s vs. 1.5 ± 0.2 m/s, *P* < 0.001). Infarcted territories had higher velocities when compared with the corresponding territories in controls: right coronary artery: 1.9 ± 0.7 m/s vs. 1.4 ± 0.3 m/s, *P* < 0.05; circumflex artery: 3.1 ± 1.5 m/s vs. 1.7 ± 0.4 m/s, *P* < 0.01; and left anterior descending artery: 1.8 ± 0.5 m/s vs. 1.4 ± 0.2 m/s, *P* < 0.01. There was a strong correlation between global MW velocities and wall motion score index (*r* = 0.70, *P* < 0.001). MW velocities were higher in segments with wall motion abnormalities than in healthy segments (2.3 ± 1.1 vs. 1.6 ± 0.7 m/s, *P* < 0.001).

**Conclusion:**

Estimation of MW velocities using 3D HFR echocardiography had excellent feasibility. MW velocities were higher in patients with AMI than in controls, in infarcted compared with healthy territories, and in segments with wall motion abnormalities. Future work should evaluate the clinical value in larger populations.

## Graphical Abstract

**Graphical Abstract:**
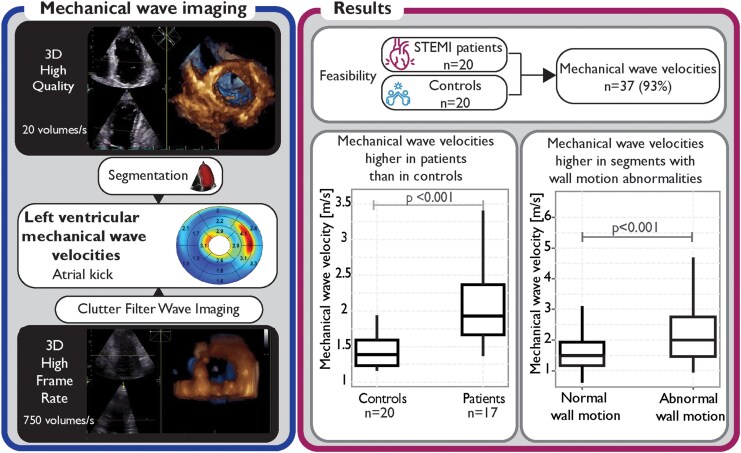
Feasibility and velocity estimation using 3D high frame rate mechanical wave imaging in patients with ST-elevation myocardial infarction. STEMI, ST-elevation myocardial infarction.

## Introduction

In patients with acute myocardial infarction (AMI), improved characterization of myocardial tissue properties may support clinical decision-making and contribute to improved patient outcomes.^1^ Echocardiography is widely available, allows for real-time imaging, and provides prognostic information that can guide treatment. However, although echocardiographic measures such as wall motion score (WMS) and myocardial strain have the potential to identify deformation abnormalities,^2^ current methods have limited ability to characterize myocardial tissue properties. Less available and more resource-demanding methods, such as magnetic resonance imaging, are therefore often required. Recently, high frame rate (HFR) echocardiography has allowed studying fundamental characteristics of the myocardium in much more detail. Of particular interest are mechanical waves (MWs), which propagate through the myocardium at high velocities.^3,4^ These waves may be externally applied, but are also naturally occurring, such as by closure of the mitral or aortic valves, or by contraction of the atrium, the atrial kick.

The velocity of MW propagation is dependent on the tissue in which they propagate and increases with increasing tissue stiffness.^5^ It has been hypothesized that MW can provide valuable insights into inherent properties of the myocardium.^6–8^ In the early phases of AMI, when the myocardium is characterized by oedema and inflammation, studies have indicated that MW velocities are higher in infarcted regions.^5,9,10^ These studies were limited to animal models or were using 2D echocardiography. However, MWs propagate through the heart in three dimensions.^8,11^ Assessment of MW by 3D HFR echocardiography is therefore a compelling method to allow easily available tissue characterization but has until now been restricted by technical challenges. To solve these challenges, we have developed a method to acquire 3D HFR images (750 volumes/s) and an advanced filter (clutter filter wave imaging), to create a 3D map of myocardial MW velocities.^8^ Our aims were to assess the feasibility of estimating MW velocities by 3D HFR and to compare MW velocities in patients with AMI to controls.

## Methods

### Study design

We included 20 patients with first-time ST-elevation myocardial infarction (STEMI) admitted to St. Olav’s University Hospital, Trondheim, Norway. Patients were included after reperfusion therapy by pharmacologic thrombolytic therapy or primary percutaneous coronary intervention (PCI). Exclusion criteria were (i) haemodynamic instability, (ii) ongoing atrial fibrillation, (iii) any previously known heart disease, and (iv) inability to consent. All patients underwent echocardiographic examinations within 48 h after admission. Coronary angiography was performed in all patients, and the infarct-related artery (IRA) was identified. Clinical variables, laboratory findings, and angiographic results were retrieved from the patient’s medical journal. The sample size was based on feasibility considerations and prior experience with similar studies, rather than a formal power calculation.

### Controls

A cohort comprising 20 healthy volunteers was randomly selected from a separate study included at the same hospital, after matching for age, gender, and body mass index (BMI).^3^ These controls were recruited through announcements on the official web pages of the Norwegian University of Science and Technology and St. Olav’s University Hospital, both located in Trondheim, Norway. Cardiac magnetic resonance imaging was performed to verify healthy myocardium in all controls.^3^

### Echocardiographic acquisitions

**Figure 1:**
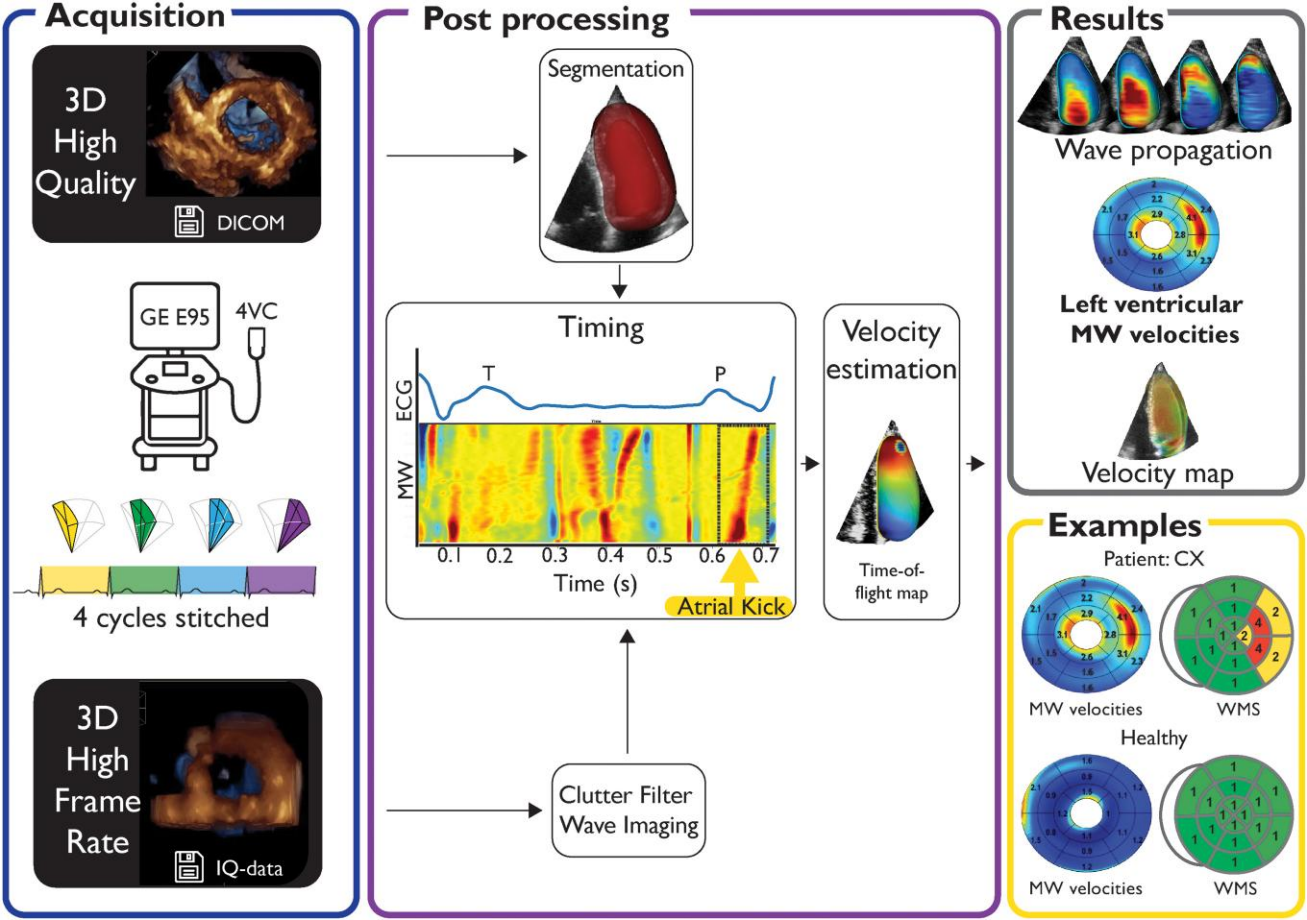
Image acquisition and processing of 3D MW velocities. A 3D HQ image was used to provide a myocardial mask. Clutter filter was then applied to the 3D HFR recording. The atrial kick wave was determined by manually identifying the anatomical M-mode signal. Velocity estimations were performed by generating a time-of-flight map, from which both direction and velocity of wave propagation were calculated. 4VC, 4Vc-D probe; CX, circumflex artery; DICOM, Digital Imaging and Communications in Medicine; ECG, electrocardiogram; GE E95, Vivid E95 scanner; IQ data, data that provide amplitude and phase of a signal; MW, mechanical wave; WMS, wall motion score.

Comprehensive clinical echocardiograms were acquired by experienced cardiologists (T.E., >3000 recordings; B.G., >10 000 recordings) using a Vivid E95 scanner with a 4Vc-D probe (GE Healthcare, Horten, Norway) at the European Association of Cardiovascular Imaging (EACVI) accredited echocardiographic laboratory at St. Olav’s University Hospital. 3D HFR MW measurements were then obtained in two steps: First, 3D high-quality (HQ) images were acquired to provide context. Second, 3D HFR images were acquired with the probe in the same position. Both 3D acquisitions were recorded with a sector width of 65˚. The HQ acquisitions had a mean frame rate of 20 ± 3 volumes/s. To achieve a HFR of at least 750 volumes/s for the 3D HFR acquisition, five steered plane waves were emitted to reconstruct a smaller volume of the left ventricle (LV). By stitching four of these volumes over consecutive cardiac cycles, the whole LV was reconstructed in three dimensions (*Figure 1*). The stitching was visually verified by looking for discontinuities in the wave propagation and time-of-flight maps. The setup is described in depth by Salles *et al.*^8^ 3D HFR acquisitions were saved as in-phase and quadrature (IQ) data for post-processing. IQ data, providing information about both amplitude and phase of the signal, were used for subsequent processing.

### Echocardiographic reference measurements

The reference acquisitions were analysed by an experienced cardiologist certified in transthoracic echocardiography by the EACVI (B.G.), using EchoPac v.204 (GE HealthCare, Horten, Norway). The reader was blinded to all clinical information. In addition to standard clinical evaluation of chamber dimensions, systolic and diastolic function, and valves, WMS and segmental peak systolic longitudinal strain by automated functional imaging were assessed. Segments with strain > −16% were categorized as pathologic.^12^ Strain analyses were performed according to recommendations from EACVI/American Society of Echocardiography (ASE), using a 16 segments LV model.^13^ Wall motion score index (WMSI) was assessed as the average WMS in a corresponding 16 segments LV model.^14^ By convention, the right coronary artery (RCA) territory comprised four segments, the left anterior descending (LAD) territory seven analysable segments, and circumflex (CX) territory five segments (see Supplementary data online, *Figure S1*).^14^ Segments with visually unacceptable image quality or poor myocardial tracking were excluded.

### 3D HFR MW imaging

Due to the HFR acquisition scheme, 3D HFR acquisitions have low image quality resulting in poor visual separation of the myocardial wall from the cavity, and separate HQ images were used for context. The segmentation mask was extracted using the 4D Auto LVQ tool in EchoPac and adapted to work with all patients (*Figure 1*). To detect MW propagation, clutter filter wave imaging was applied to the 3D HFR IQ data.^8,15,16^ The clutter filter attenuates and thus detects the Doppler spectral frequencies corresponding to the tissue velocities of the wave propagation. Measurement variance was reduced using both temporal and spatial smoothing, and the resulting data were used for visual assessment of MWs occurring during a cardiac cycle. Based on earlier studies using this method, the atrial kick wave has the best feasibility and was therefore used in this study.^8^ The duration and initiation of the atrial kick wave were set semi-automatically by considering the corresponding wave propagation on the anatomical M-mode. The electrocardiogram (ECG) was used to control the timing. The MW velocity estimations were performed by generating a 3D time-of-flight map, which is a time estimate of the wave propagation from initiation to a given point in space. From this time-of-flight map, both the 3D vector and velocity of wave propagation can be calculated (*Figure 1*).^8^ The velocity map was displayed in a 16-segment bull’s-eye plot, visualizing the average velocities for each segment. Twenty percent of the basal length and 15% of the apical length were excluded from subsequent evaluations due to the intricate geometry and the presence of valves at the ventricular base. To reduce noise, measurements were excluded in case of inverse wave propagation velocities (apex to base propagation) and for velocities outside physiologic ranges (>10 m/s). Segments lacking measurements, due to for instance dropout, in more than 50% of the segmental volume, were excluded from subsequent calculations.

### Territorial measurements

Territorial longitudinal strains and territorial MW velocities were calculated as the average of segmental values within the theoretical perfusion areas for the three main epicardial coronary arteries (see Supplementary data online, *Figure S1*).^14^

### Statistical analyses

Descriptive statistics are presented as numbers, *n* (%) for categorical variables and mean (SD) or median (IQR) for continuous variables. Normality was assessed by the Shapiro–Wilk test and visual evaluation of histograms with density plots and QQ-plots. Means were compared using Student’s *t*-tests and comparisons of medians by two-sample Wilcoxon rank-sum (Mann–Whitney) tests. The infarcted vs. non-infarcted territorial measurements were compared by a linear mixed model with subject specific random intercept and group specific residual variance, as the measurements were considered dependent. The correlations between MW velocities and global longitudinal strain (GLS), WMSI, troponin T, and LV ejection fraction (LVEF) were measured by Pearson correlation coefficient (*r*). Statistical analyses were performed by STATA/MP 18.

### Ethical approval

The study was approved by the Central Norway Regional Committee for Medical Research Ethics (nos. 204251 and 2017/1068) and the institutional personal data officer at St. Olavs Hospital/NTNU and conducted according to the Helsinki Declaration. All participants signed a written consent.

## Results

### Study population

The study population comprised 20 prospectively included patients and 20 controls, matched for age, gender, and BMI (65% male, mean age 69 years). Apart from lower heart rate and lower prevalence of smoking and hypertension in the control group, there were no significant differences in registered baseline characteristics (*Table 1*). Two patients and one control had moderate valve disease. Nine (45%) patients received pharmacologic thrombolytic therapy, of whom eight had facilitated PCI towards the IRA 11 ± 11 h after admission to hospital, and one was referred to coronary artery bypass surgery. Eleven patients (55%) received primary PCI. The RCA was the most frequent IRA, followed by the left anterior descending artery (LAD), and the circumflex artery (CX). An average troponin T level of 4021 ± 3042 ng/L among the patients reflected moderate to large infarct sizes.

**Table 1:** Study population characteristics

	Patients	Controls	*P*-value
	*n* *=* *20*	*n* *=* *20*	
Age (years), mean (SD)	69 (7)	69 (8)	0.96
Female gender, *n* (%)	6 (30)	8 (40)	0.52
BMI (kg/cm^2^), mean (SD)	26 (3)	26 (3)	0.54
Diabetes, *n* (%)	2 (10)	1 (5)	0.56
Hypertension, *n* (%)	7 (35)	1 (5)	0.02
Hypercholesterolaemia, *n* (%)	3 (15)	1 (5)	0.30
Current smoker, *n* (%)	5 (25)	0 (0)	0.02
Heart rate, median (IQR)	68 (63–76)	51 (48–58)	0.00
Thrombolysis, *n* (%)	9 (45)		
Hours from symptom onset to admission, median (IQR)	2 (2–4)		
Hours from symptom onset to reperfusion therapy^a^, median (IQR)	2 (2–3)		
Hours from admission to echo, mean (SD)	21 (9)		
Troponin T, peak value (ng/L), mean (SD)	4021 (3042)		
Infarct-related artery, *n* (%)			
Right coronary artery	11^b^ (55)		
Circumflex artery	3 (15)		
Left anterior descending artery	6^c^ (30)		

BMI, body mass index.

^a^Either primary PCI or thrombolytic treatment.

^b^One 3D HFR acquisition not feasible to analyse.

^c^Two 3D HFR acquisitions not feasible to analyse.

### Baseline echocardiographic measurements

Echocardiograms were obtained 21 ± 9 h following hospital admission. Patients with AMI had impaired global systolic function compared with controls, as evident for both LVEF, GLS, and WMSI (all *P* < 0.005; *Table 2*). There were no differences in diastolic parameters between the groups. Corresponding to the global values, there were also significant differences between patients and the controls in territorial measurements for both WMSI and longitudinal strain (*Table 2*).

**Table 2:** Echocardiographic measurements

	Patients	Controls	*P*-value
	*n* *=* *20*	*n* *=* *20*	
LVEDV (mL), mean (SD)	127 (41)	117 (24)	0.33
E/e′, median (IQR)	7.8 (6.8–10.3)	7.1 (6.4–9.6)	0.56
Mitral E velocity (m/s), mean (SD)	0.6 (0.1)	0.6 (0.1)	0.52
Mitral A velocity (m/s), mean (SD)	0.7 (0.1)	0.6 (0.1)	0.13
Mitral E/A ratio, mean (SD)	0.9 (0.3)	1.0 (0.3)	0.53
LVEF Biplane (%), median (IQR)	52 (39–57)	60 (57–62)	0.00
WMSI, median (IQR)	1.4 (1.3–1.6)	1.0 (1.0–1.0)	0.00
GLS (%), mean (SD)	−15.6 (4.5)	−19.2 (1.6)	0.00
Territorial measurements			
WMSI, RCA territory (%), median (IQR)	1.5 (1.3–1.6)	1 (1.0–1.0)	0.00
WMSI, LAD territory (%), median (IQR)	1.0 (1.0–1.6)	1 (1.0–1.0)	0.00
WMSI, CX territory (%), median (IQR)	1.4 (1.3–2.0)	1 (1.0–1.0)	0.00
Strain, RCA territory (%), mean (SD)	−12.8 (5.6)	−18.2 (2.6)	0.00
Strain, LAD territory (%), mean (SD)	−15.5 (7.6)	−19.4 (1.3)	0.03
Strain, CX territory (%), mean (SD)	−10.1 (3.0)	−12.5 (3.2)	0.02

CX, circumflex artery; LAD, left anterior descending artery; LVEDV, left ventricular end diastolic volume; LVEF, left ventricular ejection fraction; GLS, global longitudinal strain; IQR, interquartile range; RCA, right coronary artery; SD, standard deviation; WMSI, wall motion score index.

### 3D HFR MW imaging: feasibility

**Figure 2:**
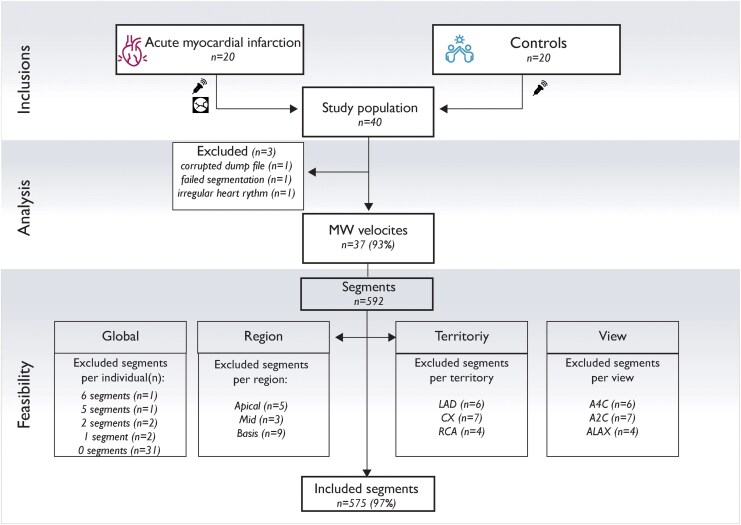
Flow chart of inclusions, analyses, and feasibility. Study flow chart. A2C, apical two-chamber view; A4C, apical four-chamber; ALAX, apical long axis; CX, circumflex artery; LAD, left anterior descending artery; MW, mechanical wave; RCA, right coronary artery.

The frame rate of the 3D HFR acquisitions was 750 volumes/s. 3D HFR MW velocities were successfully measured in 17 patients and 20 controls, resulting in a feasibility of 93% (*Figure 2*). One analysis failed in the segmentation process due to poor image quality in the HQ acquisition. In addition, one analysis failed due to technical corruption of the recording, rendering the file impossible to access and analyse. The third excluded case had extensive variation in heart rate. On a segmental level, feasibility for 3D HFR MW velocities among the included cases was 97% (575 of 592 segments). Segmental feasibilities were excellent for all territories (RCA 144/148, 97%; LAD 253/259, 98%; CX 178/185, 96%). In the RCA territory, all segments were included in 35 of 38 examinations (95%), whereas the corresponding numbers for LAD were 33 examinations (89%) and for CX 32 examinations (86%). The segmental feasibilities were similar in the apical, midventricular, and basal regions (97%, 96%, 98% accordingly) (*Figure 2*).

### 3D HFR MW imaging: velocities

**Figure 3:**
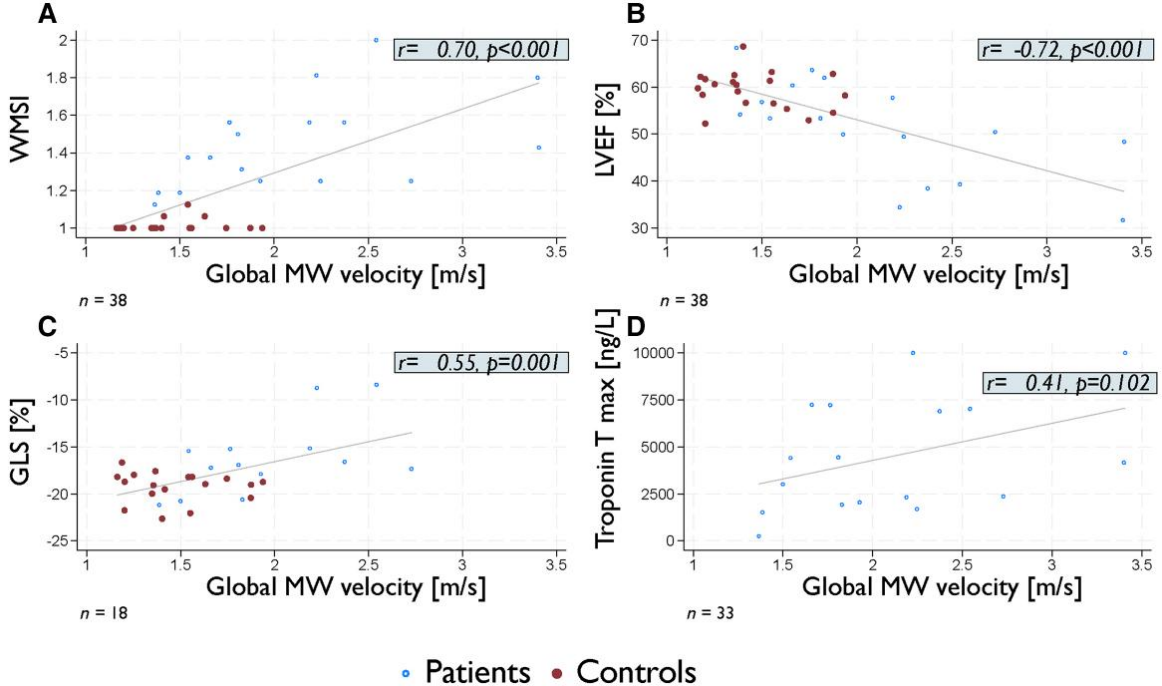
Associations between global 3D MW velocities, troponins, and global systolic parameters. Associations between the global measurements of 3D MW velocities and WMSI (*A*), LVEF (*B*), GLS (*C*), and troponin T (*D*). LVEF, left ventricular ejection fraction; GLS, global longitudinal strain; MW, mechanical wave; *r*, Pearson’s correlation coefficient; WMSI, wall motion score index.

In the total study population, mean global atrial kick MW velocity was 1.8 ± 0.6 m/s. Global MW velocities were in average 45% higher in AMI patients compared with controls [mean absolute difference 0.7 m/s, 95% confidence interval (CI) 0.3–0.9 m/s, *P* < 0.001] (*Table 3*) (*Graphical Abstract*). There was a strong correlation between global MW velocities and WMSI (*r* = 0.70, *P* < 0.001) (*Figure 3A*). Furthermore, there was a strong negative correlation between global MW velocities and LVEF (*r* = −0.72, *P* < 0.001) (*Figure 3B*). There was also a significant correlation between global MW velocities and GLS (*r* = 0.55, *P* = 0.001) (*Figure 3C*).

**Table 3:** 3D MW velocities

	Patients	Controls	Mean difference (95% CI)	*P*-value
	*n* *=* *17*	*n* *=* *20*		
Global				
3D MW velocity (m/s), mean (SD)	2.1 (0.6)	1.5 (0.2)	0.7 (0.3–0.9)	0.00
Territorial				
3D MW velocity RCA territory (m/s), mean (SD)	1.8 (0.6)	1.4 (0.3)	0.4 (0.1–0.7)	0.02
3D MW velocity LAD territory (m/s), mean (SD)	2.0 (0.6)	1.4 (0.2)	0.7 (0.4–1.0)	0.00
3D MW velocity CX territory (m/s), mean (SD)	2.4 (0.9)	1.7 (0.4)	0.8 (0.3–1.2)	0.00

CX, circumflex artery; LAD, left anterior descending artery; RCA, right coronary artery; MW, mechanical wave; SD, standard deviation.

**Figure 4:**
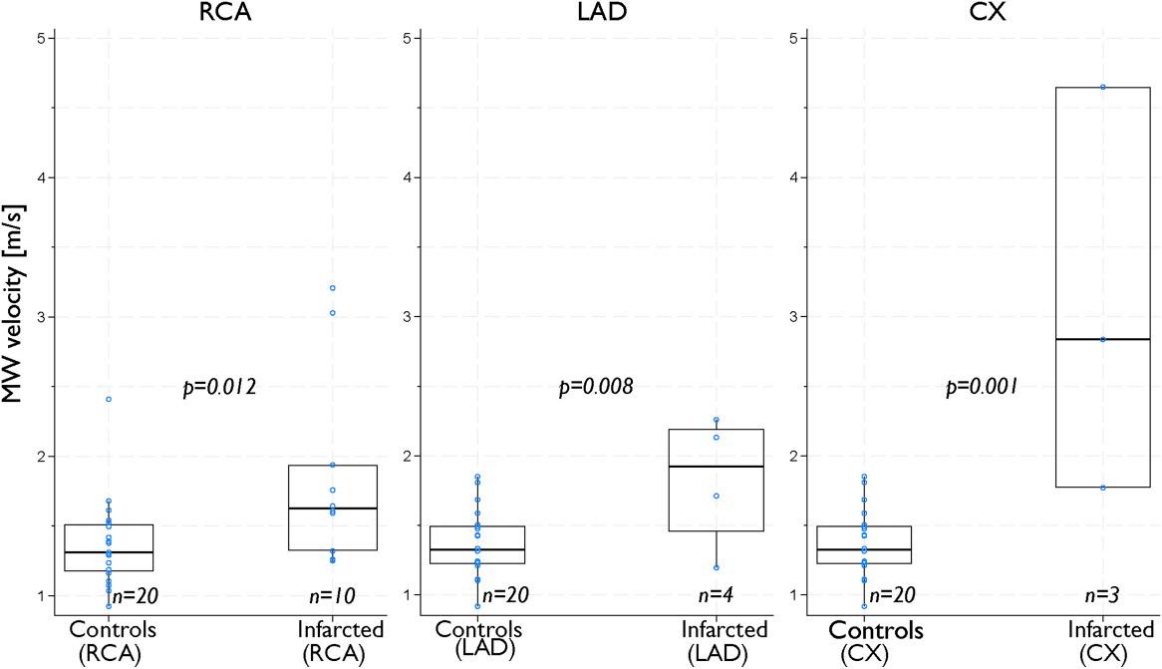
3D MW velocities in infarcted territory among patients vs. corresponding territory in controls. MW velocities in the infarct-related territory compared with the same territory in controls. CX, circumflex artery; LAD, left anterior descending artery; RCA, right coronary artery.

Corresponding to the global results, territorial MW velocities were higher in the infarcted area among patients, when compared with the same territory in controls (mean absolute difference 0.6, 95% CI 0.3–0.9 m/s, *P* < 0.01). This difference was evident for all coronary arteries (*Figure 4*). However, MW velocity measurements were available in only four patients with LAD infarction and three with CX infarction.

**Figure 5:**
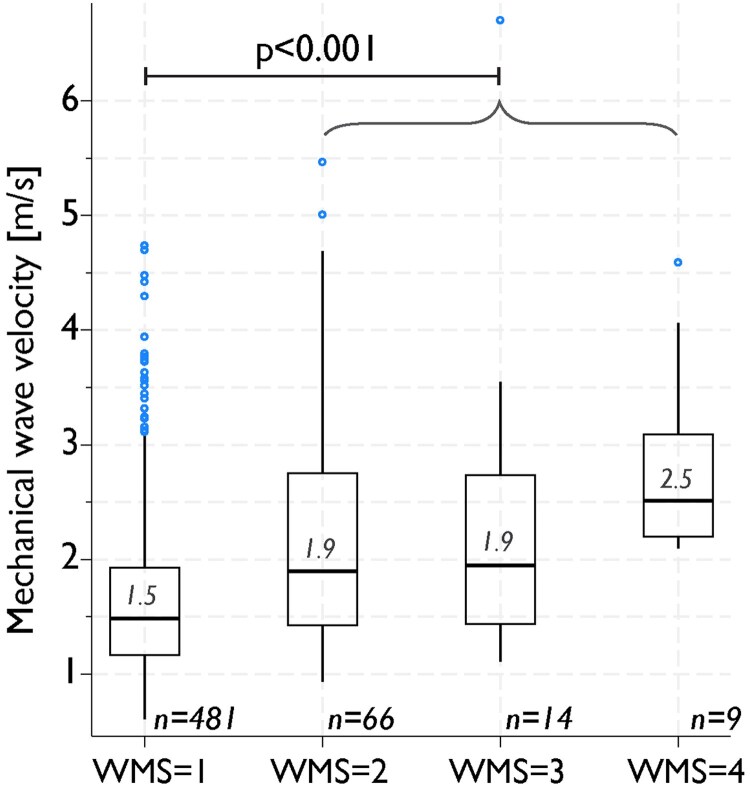
Association between segmental 3D MW velocities and WMS. 3D MW velocities categorized based on WMS: normokinetic (WMS = 1), hypokinetic (WMS = 2), akinetic (WMS = 3), and dyskinetic (WMS = 4). There was significant difference between segments with normal wall motion (WMS = 1) and segments with abnormal wall motion (WMS = 2–4). MW, mechanical wave; WMS, wall motion score.

On a segmental level, there were highly significant differences in MW velocities between segments with normal and pathologic WMS (mean absolute difference 0.7 m/s, 95% CI 0.5–0.8 m/s, *P* < 0.001) (*Figure 5* and *Graphical Abstract*). Furthermore, segments with abnormal strain values had higher MW velocities than segments with normal strain values (mean absolute difference 0.4 m/s, 95% CI 0.2–0.5 m/s, *P* < 0.001). On an individual level in patients with AMI, there were no significant differences in MW velocities between the theoretical infarct-related territory vs. the theoretical non-infarcted territories (mean absolute difference 0.0 m/s, 95% CI −0.4–0.3 m/s, *P* = 0.87).

There were no significant correlations between diastolic echocardiographic parameters and MW velocities (E/e′: *r* = −0.14, *P* = 0.42; e′ septal: *r* = 0.30, *P* = 0.72; e′ lateral: *r* = 0.21, *P* = 0.21; MV E/A ratio*: r* = 0.11, *P* = 0.542; LV end-diastolic volume: *r* = 0.38, *P* = 0.02; LV diastolic diameter: *r*  *=* 0.38, *P* = 0.54).

## Discussion

In this exploratory study, we assessed the feasibility of a novel method utilizing 3D HFR echocardiography for estimating naturally occurring atrial kick MW velocities in patients with AMI. Our main findings were the following: (i) Measurements of MW velocities using 3D HFR were highly feasible; (ii) MW velocities were higher in patients with AMI and in the infarct-related territory compared with controls; and (iii) MW velocities were higher in segments with wall motion abnormalities. As this is a pilot study, the findings cannot permit definitive conclusions regarding the underlying mechanisms, or the nature of the observed associations. Nonetheless, we believe the study highlights a promising potential of non-invasive myocardial tissue characterization by 3D HFR MW imaging and suggests focus areas for continued technical development.

### MWs and tissue properties in myocardial infarction

In the initial phase following AMI, myocardial tissue becomes oedematous with increased tissue stiffness.^17^ Animal studies have demonstrated higher MW velocities in stiffer, infarcted myocardium and showed promise in differentiation of infarcted compared with stunned myocardium.^5,9,18^ Kvåle *et al*.^10^ assessed patients in the chronic phase of myocardial infarction and demonstrated increased MW velocities, corresponding to areas with fibrotic tissue in magnetic resonance images. Our study extends these findings to the acute phase of STEMI using 3D HFR echocardiography, supporting an association between infarcted myocardium and higher MW velocities. Although MRI data were not available, the observed associations between MW velocities and WMSI, LVEF, and GLS suggest that MW velocity may serve as a marker of infarct severity. Similar to Kvåle *et al.*,^10^ we found no significant differences between infarcted and non-infarcted territories in individual patients. This may be explained by challenges related to matching of echocardiographic segments with the individual patient’s coronary perfusion areas with use of theoretical non-subject–specific 3D LV maps. Additionally, the method’s exclusion of 20% basal length and 15% apical length potentially affects the segmental comparisons. Physiological factors may also contribute, including changes in load, tethering between segments in the acute phase of a myocardial infarction, post-reperfusion stunning, remote ischaemia, and the inherent velocity differences between myocardial walls.^3,4,19^

### MW velocities by 3D HFR echocardiography

Capturing MW propagation and velocities in 3D is a breakthrough in the assessment of cardiac mechanics. To our knowledge, this is the first study to compare MW velocities obtained from 3D HFR echocardiography between patients with AMI and controls. While earlier studies have explored MW propagation using 2D HFR or in experimental models, our work extends these findings into a 3D model used in a realistic clinical setting, enabling comprehensive assessment of regional MW velocities *in vivo*.^4^ 3D-based assessment allows measuring waves that initiate and propagate out of plane in 1D and 2D and facilitates evaluation of both propagation patterns and velocities. Moreover, the angle dependency of velocity measurements is reduced. We focused on the atrial kick wave, which has the highest feasibility when using the clutter filter wave imaging.^3,8^ In contrast, waves generated by the closure of aortic and mitral valves are often not detectable or dissipate before they reach the apex. As the effect of the filter is related to the direction of wave propagation, velocities will vary depending on the imaging view and origin of MW. For MW due to valve closure and in the parasternal view, the filter may need to be modified to provide valid results. Furthermore, the atrial kick wave exhibits lower velocities compared with waves associated with aortic or mitral valve closure, which is an advantage considering the current volume rate achieved in our setup.^3^ The atrial kick wave represents a regional myocardial stretch, which may be induced by LV blood inflow propagation moving from the base to the apex, or stretch in the myocardium caused by the local volume expansion.^5,8,20–22^ This is comparable with the wave propagating in the wall of the arteries. In this situation, wave speed in the myocardium is influenced not only by stiffness but also by wall thickness and LV dimensions.^5^ During late diastole, when the LV is at its largest and wall thickness at its minimum, lower velocities are therefore expected. Furthermore, both the processing, which introduces temporal smoothing, and the exclusion of non-physiologic velocities might contribute to lower velocities when compared with previous methods.^16^

MW velocities were higher in the lateral wall of the LV than in the other regions (*Table 3*). This aligns with prior research.^3,23^ This variance in velocities between walls and also throughout the cardiac cycle warrants further investigations^24^ and may bear relevance to the boundary conditions of the heart walls, varying wave origins, loading conditions, myocyte geometry, fibre orientation, and contractility. The intricate physical nature of these waves is highly relevant for the complex interpretation of MW velocities.^24,25^

### Clinical implications and future directions

Although based on a small patient cohort, this study indicates that 3D HFR echocardiography has a potential for future clinical application, providing possible insights into the intrinsic properties of myocardial tissue traditionally explored through time-consuming, less accessible, and expensive investigations such as magnetic resonance imaging. MW assessment theoretically enables differentiation between the pathophysiological mechanisms underlying reduced myocardial deformation, potentially enhancing risk stratification and facilitating more targeted treatment strategies. The assessment of rapidly occurring cardiac events may also enhance the understanding of cardiac mechanics and in the future provide new opportunities in mapping of electromechanical activation within the cardiac cycle.^20,26^ However, integrating 3D HFR imaging in routine clinical practice necessitates advancements in technology to streamline acquisition and data handling, improve real-time quality control, and automate post-processing to achieve robust measurements of wave propagation velocities. Future research should aim to enhance feasibility of analysing natural MW caused by mitral and aortic valve closure, which have been suggested to have improved ability to separate healthy from fibrotic myocardium.^3,4^ This includes optimizing the ultrasound transmit schemes to achieve higher frame rates, to improve accuracy and feasibility in measuring the highest velocities.^4^ One way to increase frame rate further is to use broader transmit beams (e.g. diverging waves) at the expense of image quality. Diverging waves were not available on the clinical system used in this work. Also, more physiological investigations, such as wall–flow interactions, are essential for further development. Establishing consensus on MW velocity estimation methods, testing them in more extensive and diverse populations, and comparing with magnetic resonance images will be crucial for standardizing normal values and enhancing clinical utility.^4^

### Limitations

We focused only on the atrial kick wave, due to low feasibility in detecting waves after mitral and aortic valve closure with the current method. Most previous wave velocity estimations have been conducted by 2D tissue Doppler imaging, often in the parasternal long-axis view, and direct comparison of velocities is therefore challenging. Moreover, MW propagation speed may be influenced by loading conditions, an aspect that should be more comprehensively addressed in future work.^4,25,27^ Left atrial size and function were not assessed due to protocol constraints, but are unlikely to significantly influence MW velocities, which primarily reflect myocardial tissue properties. Stitching over several heart cycles may lead to misalignments when the heart rate or ECG signal is not stable over time. While we limited this artefact by aligning to the diastolic phase and manually inspected the 3D wave propagation for artefacts, patient feasibility will potentially be lower when introduced in clinical practice.

To avoid artefacts in the measurements, 20% of the basal and 15% of the apical length were excluded from the analysis. This exclusion may introduce a bias and potentially an underestimation of territorial differences, especially in the apical region of the LAD territory. Including a larger part of the ventricle would probably introduce noise and affect the feasibility. Further investigations are needed to overcome these challenges. The study was conducted in a limited cohort of patients in stable condition and in sinus rhythm, affecting the generalizability of the study. For instance, in atrial fibrillation, the atrial kick is dampened, potentially influencing both MW propagation patterns and velocities. Furthermore, the study lacks follow-up acquisitions and magnetic resonance images for direct comparison of MW velocities with fibrotic burden. Additionally, consensus on normal MW velocity range is not yet been established. This remains important areas for future research, together with assessment of inter- and intra-observer variability to ensure robustness and reproducibility of the MW velocity estimations.

## Conclusion

Measurements of MW velocities using 3D HFR echocardiography were highly feasible. MW velocities were higher in patients with AMI and in the infarct-related territory compared with controls and in segments with wall motion abnormalities. These findings demonstrate the potential of 3D HFR for enhancing MW velocity imaging and allowing for more available myocardial tissue characterization. Future work should focus on refining the technology and further evaluating the clinical value in larger and more diverse populations.

## Supplementary Material

qyaf060_Supplementary_Data

## Data Availability

The data underlying this study cannot be shared publicly due to limitations in ethical approval and patient consent.

## Lead author biography

Marlene Iversen Halvorsrød, MD, graduated from the Norwegian University of Science and Technology (NTNU) in 2011. She has clinical experience as a resident doctor in both radiology and internal medicine. Currently, she is a PhD candidate within the Ultrasound Group at the Department of Circulation and Medical Imaging, NTNU. Her research focuses on the application of echocardiography for the diagnosis and assessment of ischaemic heart disease.
