# Supplementary material for: Mechanical wave velocities in acute myocardial infarction: an exploratory study using three-dimensional high frame rate echocardiography
Source: Eur Heart J Imaging Methods Pract. 2025 May 15;3(1):qyaf060. doi: 10.1093/ehjimp/qyaf060 (PMC12130437; doi:10.1093/ehjimp/qyaf060)
Supplement: qyaf060_Supplementary_Data [file qyaf060_supplementary_data.pdf]

Supplementary material:

Supplementary figure 1: Theoretical coronary artery distribution

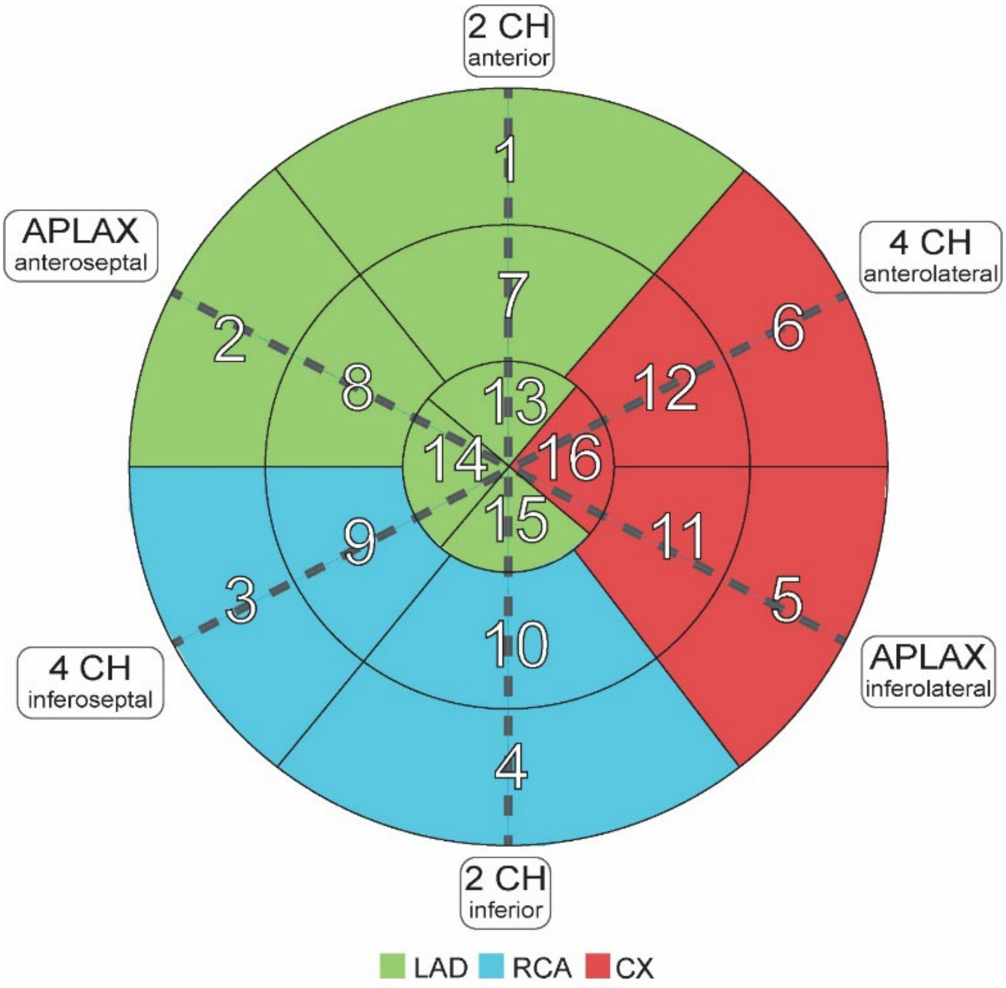

16-segment model adapted from American Heart Association (AHA) (1).

References:

1. Lang RM, Badano LP, Mor-Avi V et al (2015) Recommendations for cardiac chamber quantification by echocardiography in adults: an update from the American Society of Echocardiography and the European Association of Cardiovascular Imaging. Eur. Heart J. Cardiovasc. Imaging 16:233-270. https://doi.org/10.1093/ehjci/jev014
